# Integrated Analysis Highlights the Immunosuppressive Role of TREM2^+^ Macrophages in Hepatocellular Carcinoma

**DOI:** 10.3389/fimmu.2022.848367

**Published:** 2022-03-14

**Authors:** Lisha Zhou, Meiling Wang, Hanrui Guo, Jun Hou, Yingna Zhang, Man Li, Xiangwei Wu, Xueling Chen, Lianghai Wang

**Affiliations:** ^1^ NHC Key Laboratory of Prevention and Treatment of Central Asia High Incidence Diseases, The First Affiliated Hospital, School of Medicine, Shihezi University, Shihezi, China; ^2^ Department of Immunology, School of Medicine, Shihezi University, Shihezi, China; ^3^ State Key Laboratory of Pharmaceutical Biotechnology, Chemistry and Biomedicine Innovation Center, Department of Biotechnology and Pharmaceutical Sciences, School of Life Sciences, Nanjing University, Nanjing, China; ^4^ Key Laboratory of Xinjiang Endemic and Ethnic Diseases, School of Medicine, Shihezi University, Shihezi, China; ^5^ Department of Pathology, The First Affiliated Hospital, School of Medicine, Shihezi University, Shihezi, China

**Keywords:** HCC, scRNA-seq, Treg, NR1H3, prognosis

## Abstract

Recently, attention has been focused on the central role of TREM2 in diverse pathologies. However, the role of TREM2 signaling in the tumor microenvironment of hepatocellular carcinoma (HCC) remains poorly understood. Herein, we systematically investigated the single-cell transcriptomes of human HCC tissues and found that *TREM2* was predominantly expressed by a macrophage subpopulation enriched in tumor tissues that resemble lipid-associated macrophages (LAMs). The accumulation of TREM2^+^ LAM-like cells in HCC was confirmed in two additional cohorts using scRNA-seq analysis and immunohistochemistry. High expression of TREM2 correlated with high infiltrating macrophage abundance and poor prognosis. Based on systematic interrogations of transcriptional profiles and cellular interactions, *TREM2^+^* LAM-like cells were identified to mainly originate from *S100A8* ^+^ monocytes and represented an immunosuppressive state. TREM2^+^ LAM-like cells recruited suppressive Treg cells, facilitating microenvironment remodeling. Furthermore, gene regulatory analysis and *in vitro* functional assays indicated that activation of LXR signaling could promote the reprogramming of *TREM2* ^+^ LAM-like cells. Correlation analysis of bulk RNA-sequencing data demonstrated that the enrichment of *TREM2* ^+^ LAM-like cells was an independent indicator of adverse clinical outcomes in HCC patients. Our comprehensive analyses provide deeper insights into the immunosuppressive role of TREM2^+^ LAM-like cells in HCC.

## Introduction

Liver cancer is the third leading cause of cancer-related deaths worldwide, and hepatocellular carcinoma (HCC) accounts for 70% to 85% of the total liver cancer burden (1). Surgical resection represents the most effective treatment with curative potential for HCC (2). However, due to aggressive growth and late symptom presentation, most HCC patients are diagnosed at advanced stages and are not eligible for surgical treatments (3). To improve patient survival and quality of life, novel biomarkers of the prognosis and for the treatment of liver cancer urgently need to be explored (4).

Triggering receptor expressed on myeloid cells 2 (*TREM2*) encodes an innate immune receptor expressed on the surface of cells of the myeloid lineage, including monocytes, macrophages, osteoclasts, or microglia (5). TREM2 is an anti-inflammatory receptor that negatively regulates Toll-like receptor (TLR)-mediated inflammatory responses, whereas the proinflammatory receptor TREM1 augments TLR-induced inflammation (6). In recent years, the research community has focused its attention on the central role of TREM2 and myeloid cells in diverse pathologies (7). TREM2 has been extensively studied in microglia for its capacity to sustain microglial responses to neurodegenerative pathologies, such as Alzheimer’s disease (8). TREM2 promotes the optimal microglial function required to attenuate disease progression and is a potential target for eliciting a protective role of microglia in Alzheimer’s disease and other neurodegenerative diseases (8). TREM2 is also expressed in several peripheral macrophage populations involved in host defense and metabolism (9). In mouse liver, TREM2 is predominantly expressed in nonparenchymal cells, including Kupffer cells and hepatic stellate cells (10). In line with TREM2’s effects on inflammation, Kupffer cells and hepatic stellate cells isolated from *Trem2* ^-/-^ mice exhibit augmented proinflammatory responses upon TLR4 stimulation (10). Moreover, mice lacking *Trem2* display exacerbated liver damage and inflammation in the context of toxin-induced hepatocellular injuries and even an elevated tumor burden in the early phases of liver tumorigenesis, suggesting that TREM2 plays a protective role in hepatocarcinogenesis (10, 11). An unbiased single-cell RNA sequencing (scRNA-seq) approach identified a subpopulation of circulating monocyte-derived scar-associated TREM2^+^CD9^+^ macrophages (SAMs) that spatially accumulate within the fibrotic niche of cirrhotic human livers (12). Trem2- and Cd9-expressing hepatic lipid-associated macrophages (LAMs) have also been described recently in metabolic-associated fatty liver disease (13–15). However, the role of TREM2 in human cancer is poorly understood. Several studies suggest that high TREM2 expression correlates with worsened outcomes in various cancers, including gastric cancer, glioma, and renal cell carcinoma (16–18). In contrast, a recent study showed that TREM2 expression was decreased in hepatoma cells and acted as a tumor suppressor (19). Thus, the function of TREM2 in human HCC, especially in tumor immune responses, warrants further investigation.

In the current study, we systematically interrogated the single-cell transcriptomes of human HCC tissues and found that TREM2 was enriched in a macrophage subgroup derived from tumor tissues that resemble LAMs. The presence of these cells and the prognostic significance of TREM2 expression were confirmed in independent cohorts. Comparative analyses of cell trajectories, cellular interactions, and gene regulatory networks led to the identification of a novel immunosuppressive role of TREM2 in HCC. In addition, we investigated the prognostic value of *TREM2* ^+^ LAM-like cells by correlating the scRNA-seq data with bulk RNA sequencing (RNA-seq) profiles from publicly available datasets. These results will help elucidate the biological significance of TREM2 and promote the improvement of clinical treatment strategies for HCC patients.

## Materials and Methods

### Data Acquisition

The human HCC scRNA-seq data described by Sharma et al. (20) were derived from Seurat integration of fetal liver, adjacent liver, and tumor cells from HCC patients (n=14) and were downloaded from https://data.mendeley.com/datasets/6wmzcskt6k/1 as the Discovery cohort. Droplet-based expression profiles of single CD45^+^ immune cells from five HCC patients (Validation cohort 1) were obtained from the Gene Expression Omnibus (GEO) database under accession number GSE140228 (21), and data from tumor and adjacent liver tissues were extracted according to tissue site. Gene expression and clinical information from The Cancer Genome Atlas Liver Hepatocellular Carcinoma (TCGA-LIHC) project, comprising 374 tumors and 50 normal samples, were downloaded from the Genomic Data Commons (GDC) Data Portal (https://portal.gdc.cancer.gov/). Patients with a follow-up time of 0 days were excluded from the survival analysis. A liver cancer microarray dataset containing 225 HBV-related HCC and 220 nontumor tissues under accession number GSE14520 (GPL3921 Affymetrix HT Human Genome U133A Array) was obtained.

### Single-Cell RNA Sequencing Data Processing

To construct a global HCC atlas, the Seurat R package (v4.0.0) (22) was used for quality control of single cells, data normalization, dimension reduction, and unsupervised clustering. Specifically, cells with less than 200 unique molecular identifier (UMI) counts or over 10% mitochondrial UMIs were considered low-quality cells and removed. The normalization methods LogNormalize and SCTransform with default parameters were adopted for the human HCC scRNA-seq data described by Sharma et al. and the GSE140228 dataset, respectively. The RunPCA function was applied to reduce the dimensionality of the datasets. Subsequently, the main cell clusters were identified using the FindClusters function (resolution = 0.8). Significant principal components were utilized for unsupervised Louvain clustering and visualized using uniform manifold approximation projection (UMAP) with default parameters.

### Immunohistochemical Staining

Formalin-fixed paraffin-embedded tissue blocks from 113 HCC patients (Validation cohort 2) who underwent surgical resection without prior radiotherapy or chemotherapy between 2011 and 2019 were obtained from the First Affiliated Hospital, Shihezi University School of Medicine. Informed consent was obtained from the patients, and the study was approved by the Ethics Committee of the First Affiliated Hospital, Shihezi University School of Medicine. Tissue microarrays containing tumor specimens and corresponding noncancerous liver tissues were cut into 4-μm sections, dewaxed, and rehydrated. After heat-mediated antigen retrieval in EDTA buffer (pH 8.0) for 10 min using a pressure cooker, sections were rinsed with PBS and treated with 3% H_2_O_2_ to block endogenous peroxidase. Sections were blocked using 10% normal goat serum (ZLI-9022, ZSGB-BIO) and incubated with anti-TREM2 antibody (#91068, 1:1000, Cell Signaling Technology) overnight at 4°C, followed by secondary antibody incubation for 30 min at 37°C. Color was developed using 3,3-diaminobenzidine and counterstaining with hematoxylin. The counts for TREM2^+^ cells represent an average number of positively stained cells within three random intratumor areas under a 20X objective, as evaluated by experienced pathologists who were blinded to the clinicopathologic data.

### Multiplex Immunofluorescence Staining

Multiplex staining was performed using a multiplex fluorescent immunohistochemistry staining kit (Yuanxibio) according to the manufacturer’s instructions with the following primary antibodies/fluorescent dyes: TREM2 (#91068, 1:1000, Cell Signaling Technology)/Neon-TSA620, FoxP3 (MAB8214, 1:200, R&D Systems)/Neon-TSA520, and CD163 (ab182422, 1:300, Abcam)/Neon-TSA670. Primary antibodies were sequentially applied, followed by horseradish peroxidase-conjugated secondary antibody incubation (DS9800, Lecia Biosystems) and tyramide signal amplification. Sections were then counterstained with DAPI and scanned using the Pannoramic MIDI imaging system (3D HISTECH).

### Pseudotime Trajectory Analysis

The Monocle3 R package (v0.2.1), an unsupervised algorithm that increases the temporal resolution of transcriptome dynamics (23), was used to infer the cell lineage trajectories of monocyte and macrophage subsets, with parameter num_dim = 50. The dimensionality of the data was reduced, and the cells were plotted and colored by pseudotime to visualize how the cell lineages varied along the trajectories of the UMAP plots using the plot_cells function. Dynamics of gene expression within the trajectories were visualized with the plot_genes_in_pseudotime function.

### Pathway Analysis

Gene set enrichment analysis (GSEA) (24) was applied using 50 hallmark gene sets (h.all.v7.4.symbols.gmt) to identify significantly enriched functional pathways *via* GSEA software (v4.1.0), with screening criteria of nominal *P*-value < 0.05 and false discovery rate (FDR) q value < 0.25.

The functional phenotypes of each macrophage subset were defined using gene set variation analysis (GSVA) with the GSVA package (25). The gene signatures used for GSVA analysis are listed in **Supplementary Table 1**. The angiogenic and phagocytic signatures were described by Cheng et al. (26) in the supplemental data. The gene sets associated with M1/M2 polarization were described by Azizi et al. (27). The metabolic signatures of the TCA cycle, lipolysis, and glutamine metabolism were obtained from PathCards (https://pathcards.genecards.org/).

### Cell-Cell Interaction Analysis

CellPhoneDB (https://www.cellphonedb.org), a Python-based tool for systematic analysis of cell-cell communication networks, was used to infer ligand-receptor interactions among *TREM2* ^+^ LAM-like cells, T cells, and endothelial cells in adjacent liver and tumor tissues. Ligand-receptor interactions between two cell clusters were identified based on the specific expression of a receptor by one cell population and a ligand by another cell type. Interaction pairs with *P*-value < 0.05 were selected. Circle plots illustrating the interactions between cell types were drawn using the chordDiagram function from the circilize package (v0.4.12).

CellChat, another tool that can quantitatively infer and analyze intercellular communication networks from scRNA-seq data, delineates the specific signaling roles played by each cell type (28). The computeCommunProbPathway function was used to compute the communication probability at the signaling pathway level by summarizing all related ligands/receptors with default parameters. The contribution of each ligand-receptor pair to the overall signaling pathways was computed and visualized using the netAnalysis_contribution function.

### Gene Regulatory Network Analysis

SCENIC, a computational workflow that predicts transcription factor (TF) activities from scRNA-seq data (29), was used to construct gene regulatory networks (GRNs) in myeloid cell subsets from human HCC samples with default parameters, and the normalized data matrix from Seurat was used as input. Specifically, the output from GENIE3/GRNBoost was converted to coexpression modules, and the runGenie3 function with parameter nParts = 20 was used to infer gene coexpression networks. To prune coexpression modules, TF motif enrichment analysis with RcisTarget was utilized to identify TF binding motifs. Regulons in individual cells were scored by AUCell with the runSCENIC_3_scoreCells function.

SEEK analysis (https://seek.princeton.edu/seek/), a tool that navigates the massive human expression compendium (30), was applied to validate that the predicted regulons were functionally related to their associated cell types. The human version of SEEK was used to evaluate whether the genes in a regulon were coexpressed in a given cell type. It could be inferred that the function of a regulon was highly associated with a cell type if the genes were significantly coexpressed in many datasets related to the specific cell type.

### LXR Agonist Stimulation and Gene Silencing

Authenticated human monocytic THP-1 cells were purchased from the Cell Bank, Type Culture Collection, Chinese Academy of Sciences. THP-1 cells were maintained in RPMI-1640 medium (Gibco) supplemented with 10% FBS, 0.05 mM 2-mercaptoethanol, and 1% penicillin-streptomycin at 37°C in a humidified 5% CO_2_ atmosphere. THP-1 cells (2×10^5^ per well) were seeded in 12-well plates and differentiated using 50 ng/ml phorbol 12-myristate-13-acetate (PMA; CS0001, Multi Sciences) for 48 hours to obtain macrophage-like cells. Then, the cells were treated with the LXR agonists GW3965 and T0901317 (Selleck Chemicals) at 1 μM for 24 hours. For gene silencing, differentiated THP-1 cells were transfected with small interfering RNA (siRNAs) targeting *NR1H3* (LXR-α), *HIF1A* (HIF-1α), or scramble control (GenePharma) with GP-transfect-Mate (GenePharma). Oligonucleotide sequences for gene knockdown are listed in **Supplementary Table 2**. All experiments were performed with mycoplasma-free cells.

### qRT-PCR

Total RNA was extracted using the E.Z.N.A. Total RNA Kit I (Omega Bio-tek). Complementary DNA was synthesized using a RevertAid First Strand cDNA Synthesis Kit (Thermo Scientific). Gene expression levels were detected using the UltraSYBR Mixture (CWBIO). The relative expression levels of the target genes were normalized to that of β-actin using the 2^-ΔΔCt^ method.

### Deconvolution of Bulk RNA-Seq Data

The relative abundance of infiltrating immune cell types, estimated using CIBERSORT with a signature matrix containing 22 functionally defined immune subsets (LM22), in each tumor sample from the TCGA-LIHC dataset was obtained from https://gdc.cancer.gov/about-data/publications/panimmune. To evaluate the clinical relevance of the macrophage clusters identified in the present study, a custom signature matrix was first constructed by CIBERSORTx (31) from the scRNA-seq data of myeloid cells derived from the Discovery cohort (summarized as transcripts per million) with Single Cell Input Options = 0. Based on the signature matrix, the macrophage composition of each tumor sample was deconvolved with S-mode batch correction.

Enrichment scores of 24 immune cell types, for which marker genes were extracted from Bindea et al. (32), were calculated using single-sample gene set enrichment analysis (ssGSEA) implemented in the GSVA R package with default parameters.

### Statistical Analysis

Comparisons of changes between two groups were performed using nonparametric paired or unpaired Wilcoxon tests or parametric Student’s *t*-tests. Kaplan-Meier analysis and Cox hazard regression analysis were used to estimate patient survival using the survival and survminer R packages. *P* < 0.05 was considered statistically significant.

## Results

### Overview of Single Cells Derived From HCC and Nonmalignant Tissues

**Figure 1:**
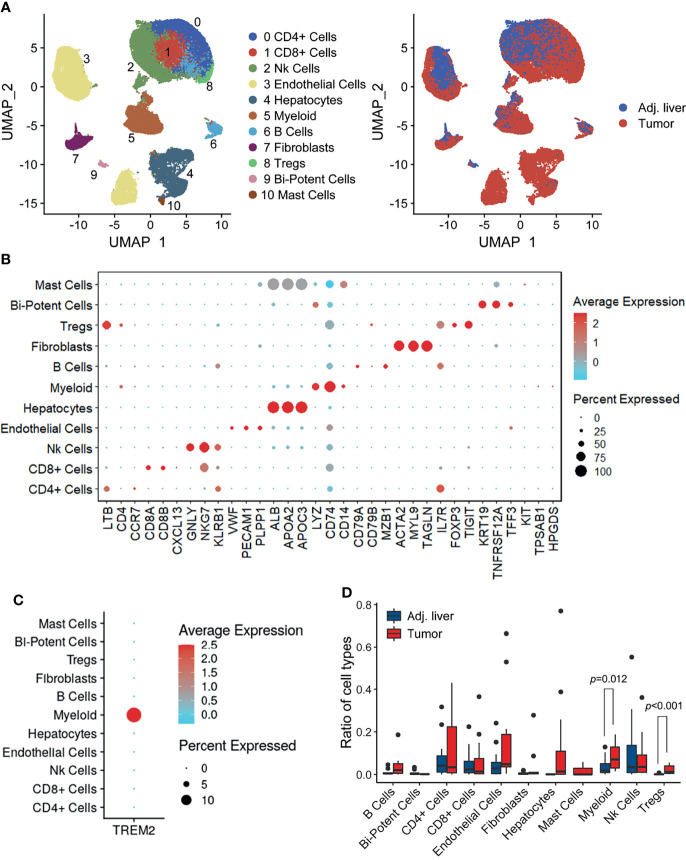
Overview of infiltrating cell types in HCC. **(A)** UMAP projection of single cells derived from 14 HCC patients colored by cell clusters (left) and tissue origin (right). **(B)** Bubble heatmap depicting the expression levels of cluster-specific marker genes. The dot size indicates the fraction of expressing cells, and dots are colored based on average expression levels. **(C)** Dot plot showing the expression levels of *TREM2* in the major cell types. **(D)** Box plot illustrating the fractions of the indicated cell types (divided by the total cell number) in adjacent liver and tumor tissues. Significance was determined using unpaired Wilcoxon test.

To explore the complexity of TREM2^+^ cells in the tumor microenvironment (TME), we first characterized the human HCC single-cell transcriptome atlas of tumor and corresponding nontumor samples from 14 HCC patients (20) (Discovery cohort). After quality control and removal of batch effects, a total of 73,564 cells were subjected to downstream analyses. We identified and visualized 11 major cell clusters through UMAP plots (**Figure 1A**). Cell type-specific markers for each cluster were identified based on the top differentially expressed genes and used to annotate cell types (**Figure 1B**). Among all the major cell types, we found that *TREM2* was mainly expressed in myeloid cells, the proportion of which was significantly higher in tumor samples than in adjacent liver tissues (**Figures 1C, D**). These findings suggested a central role of myeloid cells in TREM2-mediated immune signaling.

### Identification of the Tumor-Infiltrating TREM2^+^ Macrophage Subtype

**Figure 2:**
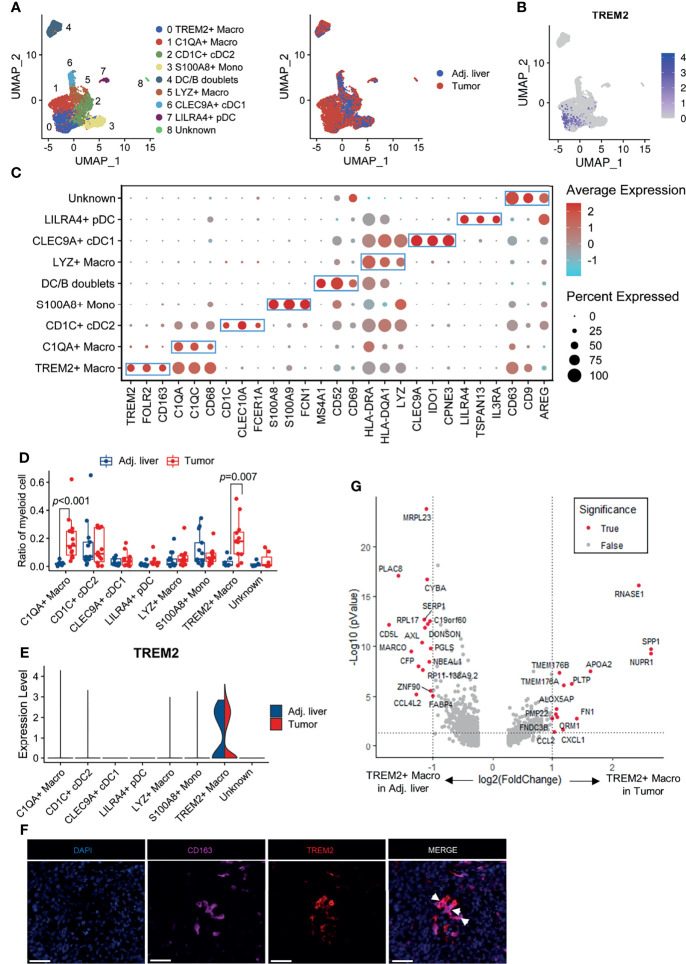
TREM2 expression defines a tumor-infiltrating myeloid subpopulation. **(A)** UMAP projection showing the subtypes of myeloid cells colored by cluster (left) and tissue origin (right). **(B)** UMAP plot of myeloid cells colored by *TREM2* expression level. **(C)** Bubble heatmap depicting the expression of marker genes in the indicated subtypes of myeloid cells. The dot size indicates the fraction of expressing cells, and the dots are colored based on average expression levels. **(D)** Box plot representing the fraction of myeloid subgroups in tumor and adjacent liver tissues. Significance was determined by unpaired Wilcoxon test. **(E)** Violin plot showing the expression level of *TREM2* in myeloid subgroups derived from tumor and adjacent liver tissues. **(F)** Representative images of immunofluorescence staining of TREM2 and CD163 in HCC tissues, showing the infiltration of TREM2^+^CD163^+^ macrophages. The scale bar represents 50 μm. **(G)** Volcano plot showing differentially expressed genes between *TREM2* ^+^ macrophages derived from tumor samples and those derived from adjacent liver tissues. Significance was determined by Benjamini-Hochberg adjusted two-sided Wilcoxon test.

Subsequently, we set out to identify the TREM2-expressing cell subtypes in the myeloid cell lineage. Further clustering of myeloid cells gave rise to 9 subpopulations representing monocytes, macrophages, and dendritic cells (cells resembling doublets were removed from further analyses; **Figure 2A**). We noticed that *TREM2* was expressed almost exclusively in tumor-derived cells (**Figure 2B**). The *TREM2* ^+^ macrophage subpopulation was characterized by the high expression of the marker genes *TREM2*, *FOLR2*, and *CD163* (**Figure 2C**). Notably, as the gene expression profile of *TREM2* ^+^ macrophages best resembled those of recently described hepatic LAMs (**Supplementary Figure 1**), we referred to this subset as *TREM2* ^+^ LAM-like cells. In addition, *TREM2* ^+^ LAM-like cells were predominantly enriched in tumor compared with adjacent nontumor tissues, although displayed a comparable expression level of TREM2 (**Figures 2D, E**). To verify the findings from single-cell analysis in the Discovery cohort, we collected the scRNA-seq profiles of tumor and adjacent liver tissues from five additional HCC patients (21) (Validation cohort 1). The results confirmed the preferential enrichment of the *TREM2* ^+^ LAM-like cells subpopulation in tumor samples (**Supplementary Figures 2A–F**), demonstrating the rationality of our cluster identification. To confirm the presence of TREM2^+^ LAM-like cells, we also performed dual-color immunofluorescence staining and found that TREM2^+^CD163^+^ macrophages were highly enriched in HCC tissues (**Figure 2F**). Differentially expressed gene analysis identified *SPP1* and *CCL2* as significantly upregulated genes in those *TREM2* ^+^ LAM-like cells derived from tumor samples compared to those derived from adjacent liver tissues (**Figure 2G**), in line with reports stating that SPP1 is secreted by infiltrating macrophages and stimulates angiogenesis (33) and that CCL2 participates in tumor-mediated immunosuppression by recruiting immunosuppressive Treg cells and myeloid-derived suppressor cells into the glioblastoma microenvironment (34).

### High Expression of TREM2 Correlates With High Infiltrating Macrophage Abundance and Poor Prognosis

**Figure 3:**
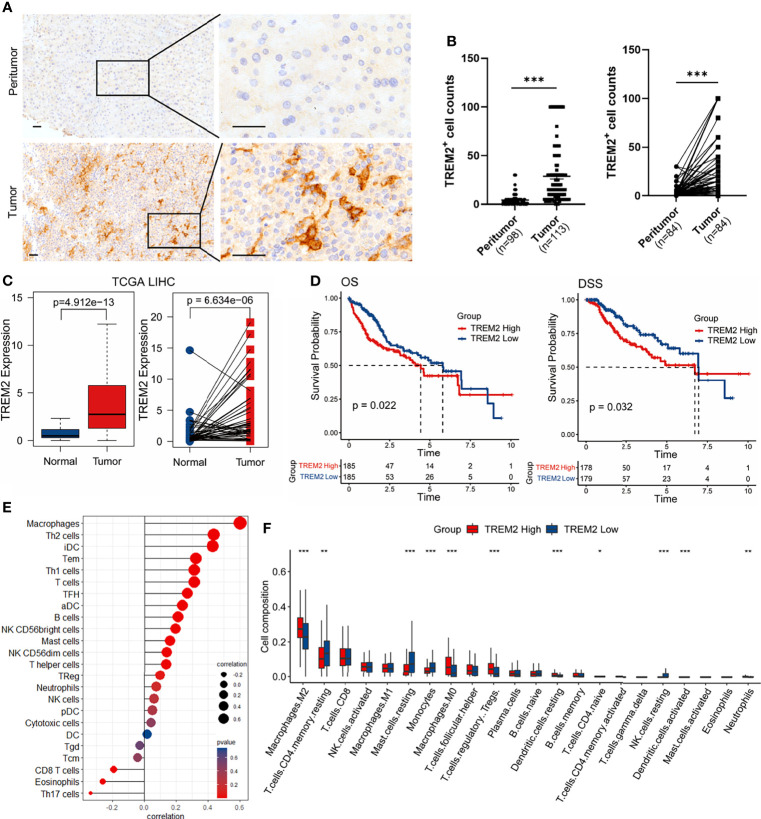
TREM2 is upregulated in HCC and correlates with worse outcomes. **(A)** Representative images of immunohistochemical staining of TREM2 expression in HCC samples and peritumor tissues. The scale bar represents 50 μm. **(B)** Statistical analysis of TREM2^+^ cell counts in all (left) or paired (right) tumors and peritumor tissues from Validation cohort 2. Significance was determined by unpaired or paired Wilcoxon tests. **(C)** Box plots comparing *TREM2* expression between all (left) or paired (right) tumor and normal samples from the TCGA-LIHC cohort. *P* values were determined by unpaired or paired Wilcoxon test. **(D)** Kaplan-Meier curves for overall survival (OS) and disease-specific survival (DSS). Patients were divided into high and low *TREM2* expression groups using the median value as the cutoff. *P* values were calculated using the log-rank test. **(E)** Lollipop plot depicting Spearman’s correlation coefficients between *TREM2* expression and 24 immune cell types calculated by ssGSEA in the TCGA-LIHC cohort. **(F)** Box plot illustrating the infiltration of 22 immune cell types estimated by CIBERSORT in patients with high and low *TREM2* expression levels. Significance was determined by unpaired Wilcoxon test. *P < 0.05, **P < 0.01, ***P < 0.001.

To address the significance of our findings that TREM2 was predominantly expressed by a macrophage subpopulation enriched in tumor tissues, we first analyzed TREM2 protein expression by immunohistochemical staining of tissue microarrays containing matched adjacent liver and tumor specimens obtained from 113 HCC patients (Validation cohort 2). The accumulation of TREM2^+^ cells was confirmed in virtually all tumor samples, while TREM2 staining was scant or absent in adjacent liver tissues (**Figure 3A**), suggesting that TREM2 may be a particularly attractive therapeutic target. The number of TREM2^+^ cells in HCC tissues was significantly higher than that in adjacent liver tissues (**Figure 3B**). We next explored the association between TREM2 expression and clinical outcomes in patients from publicly available datasets. Upregulated *TREM2* expression levels in liver cancer compared to nontumor tissues were validated in the TCGA-LIHC and GSE14520 datasets (**Figure 3C** and **Supplementary Figure 3A**). In the TCGA-LIHC cohort, *TREM2* expression correlated with inferior overall and disease-specific survival (**Figure 3D**). To infer the relationship between *TREM2* expression and immune cell characteristics, we performed Spearman’s correlation analysis of the enrichment scores of 24 immune-related cells calculated by ssGSEA. The *TREM2* expression level showed the strongest positive correlation with macrophages and Th2 cells (**Figure 3E**, **Supplementary Figure 3B**). A simple categorization system, namely classically activated M1 and alternatively activated M2 macrophage polarization, has been previously used to describe the *in vitro* activation phenotypes of macrophages (26). We also explored the association between *TREM2* expression and immune cell infiltration abundance deconvoluted by CIBERSORT to assess immune functions. A higher proportion of M2 and M0 macrophages but fewer monocytes and CD4^+^ memory resting T cells were observed in tumors with high *TREM2* expression in both the TCGA-LIHC and GSE14520 cohorts (**Figure 3F**, **Supplementary Figure 3C**).

### State Transition of TREM2^+^ LAM-Like Cells

**Figure 4:**
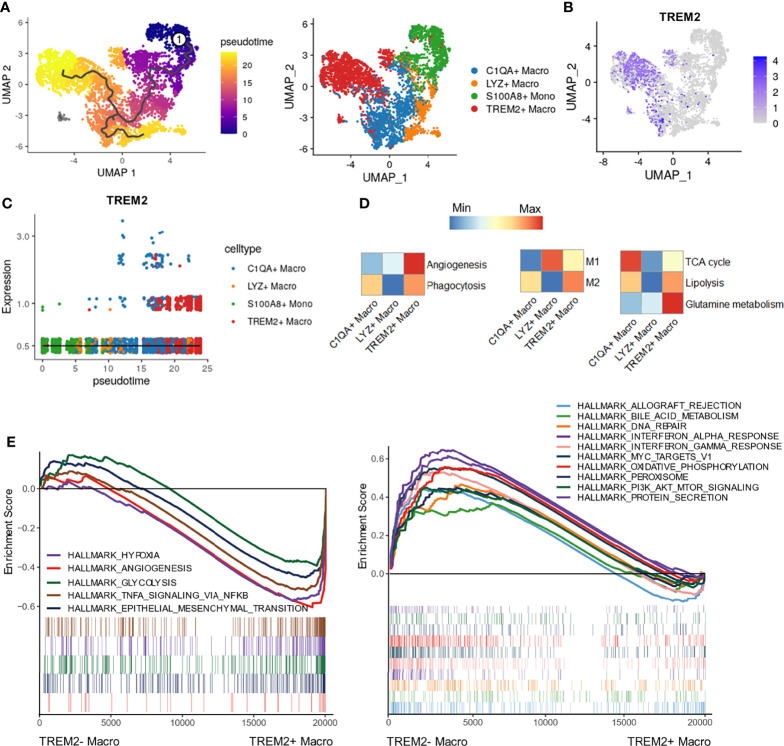
Cellular trajectory and functional phenotypes of TREM2^+^ macrophages. **(A)** Pseudotime trajectory analysis of *TREM2* ^+^ macrophages. **(B)** UMAP plot showing the expression of *TREM2* in macrophage subpopulations and monocytes. **(C)** 2D pseudotime graph showing the dynamic change in *TREM2* expression. Cell subtypes are labeled by colors. **(D)** Heatmaps showing different expression patterns of the indicated signature genes among macrophage subtypes calculated by GSVA. **(E)** Enrichment plots of significantly enriched hallmark gene sets in *TREM2* ^+^ and *TREM2* ^-^ macrophages.

To explore the cell fate transition of infiltrating TREM2^+^ LAM-like cells in HCC, we inferred cell trajectories by using Monocle3. Pseudotime analysis showed that the *TREM2* ^+^ LAM-like cells originated from *S100A8* ^+^ monocytes, which had the lowest pseudotime value, and developed through an intermediate state of *C1QA* ^+^ macrophages (**Figures 4A–C**). To delineate the functional profile of the TREM2^+^ macrophage subtype, we assessed angiogenesis/phagocytosis-, M1/M2 polarization-, and metabolism-associated signature genes. As expected, *TREM2* ^+^ LAM-like cells showed preferential expression of genes involved in angiogenesis and higher M2 polarization scores (**Figure 4D**). However, there is a limitation for the *in vitro* M1/M2 dualistic model to fully discriminate *TREM2* ^+^ LAM-like cells from *C1QA* ^+^ macrophages, suggesting more complex phenotypes of macrophages in the TME. Analysis of energy metabolism pathways showed that the *TREM2* ^+^ macrophage subtype exhibited the highest expression scores for glutamine metabolism and lipolysis (**Figures 4D**), indicating that metabolic disorders might contribute to macrophage dysfunction, consistent with glutamine promoting M2 macrophage polarization (35). GSEA revealed that genes involved in hypoxia, glycolysis, and angiogenesis were significantly enriched in *TREM2* ^+^ LAM-like cells (**Figure 4E**), which was confirmed in Validation cohort 1 (**Supplementary Figure 2G**). On the other hand, *TREM2^-^* macrophages were significantly enriched in interferon response pathways and oxidative phosphorylation pathways (**Figure 4E**), further confirming the proangiogenic phenotype and immunosuppressive state of *TREM2* ^+^ LAM-like cells.

### Interaction Network of TREM2^+^ LAM-Like Cells

**Figure 5:**
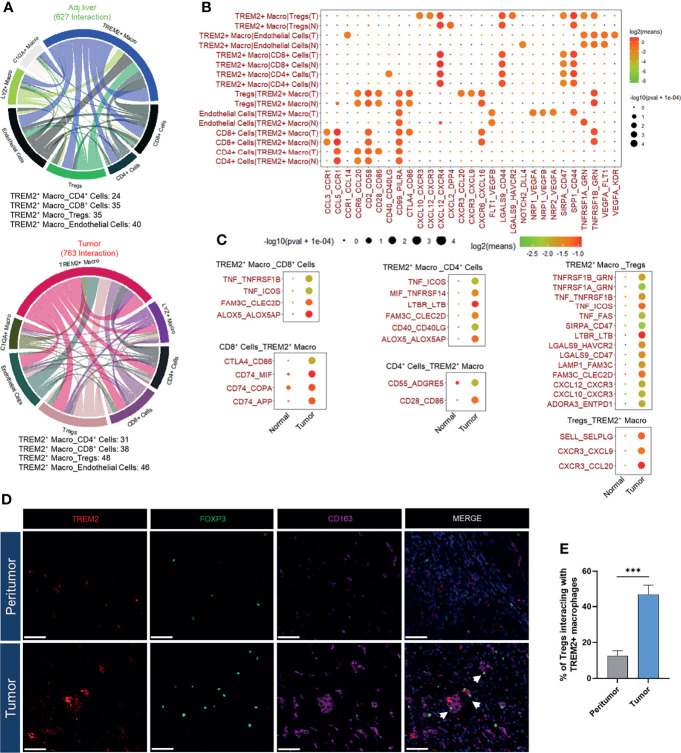
Characterization of the TREM2^+^ macrophage-based cellular interactome. **(A)** Chord diagrams of the cellular interactome between *TREM2* ^+^ macrophages, T cells, and endothelial cells in adjacent liver and tumor tissues constructed by CellPhoneDB. **(B)** Bubble plot showing ligand-receptor interactions between *TREM2* ^+^ macrophages, T cells, and endothelial cells in adjacent liver and tumor samples. Rows represent each cell-cell interaction pair, and columns define ligand-receptor pairs. The sizes of the bubbles indicate the significance of the interactions, as calculated by CellPhoneDB. **(C)** Dot plot of HCC-specific ligand-receptor interactions of *TREM2* ^+^ macrophages with T cells. **(D)** Representative immunofluorescence images of TREM2, CD163, and FOXP3 staining in HCC and peritumor tissues. Arrows denote the proximity of TREM2^+^CD163^+^ macrophages and FOXP3^+^ Treg cells. The scale bar represents 50 μm. **(E)** Quantification of FOXP3^+^ Tregs interacting with TREM2^+^ macrophages in tumors and peritumor tissues. Significance was determined by Student’s *t*-tests.

Based on the above signature analysis, we employed CellPhoneDB (36) to identify potential ligand-receptor pairs between *TREM2* ^+^ LAM-like cells, endothelial cells, and T cells, thus deciphering the intercellular communications in the HCC TME. We observed 627 ligand-receptor interactions in adjacent liver tissues and 763 interactions in tumors (**Figure 5A**). More interactions between *TREM2* ^+^ LAM-like cells and T cells (CD4^+^, CD8^+^, and Treg cells) were detected in the microenvironment of tumors than in adjacent liver tissues (**Figures 5B, C**). Interactions between *TREM2* ^+^ LAM-like cells and CD4^+^ and CD8^+^ T cells in HCC were mainly associated with immune-related ligands and receptors (CD40LG : CD40 and CD28:CD86 for CD4^+^ T cells; CD86:CTLA4 for CD8^+^ T cells). Notably, *TREM2* ^+^ LAM-like cells displayed obviously more immunosuppressive interactions with Treg cells in tumors than in adjacent liver tissues, which might dampen the function of effector T cells. *TREM2* ^+^ LAM-like cells were also found to interact with Treg cells *via* the CCL20/CXCL9/CXCL10/CXCL12–CXCR3 axes, suggesting that *TREM2* ^+^ LAM-like cells attract Treg cells *via* migration-related chemokines. These results highlight that *TREM2* ^+^ LAM-like cells can regulate multiple T cell subsets, especially by recruiting suppressive Treg cells, which may induce a compromised antitumor immune response in HCC. Remarkably, we observed that FOXP3^+^ Treg cells and TREM2^+^CD163^+^ macrophages colocalized within the tumor ecosystem by employing multicolor immunofluorescence staining (**Figures 5D, E**). Taken together, these findings demonstrate that TREM2^+^ LAM-like cells can contribute to microenvironment remodeling and facilitate an immunosuppressive tumor ecosystem.

### Identification of Specific Regulators of TREM2^+^ LAM-Like Cells

**Figure 6:**
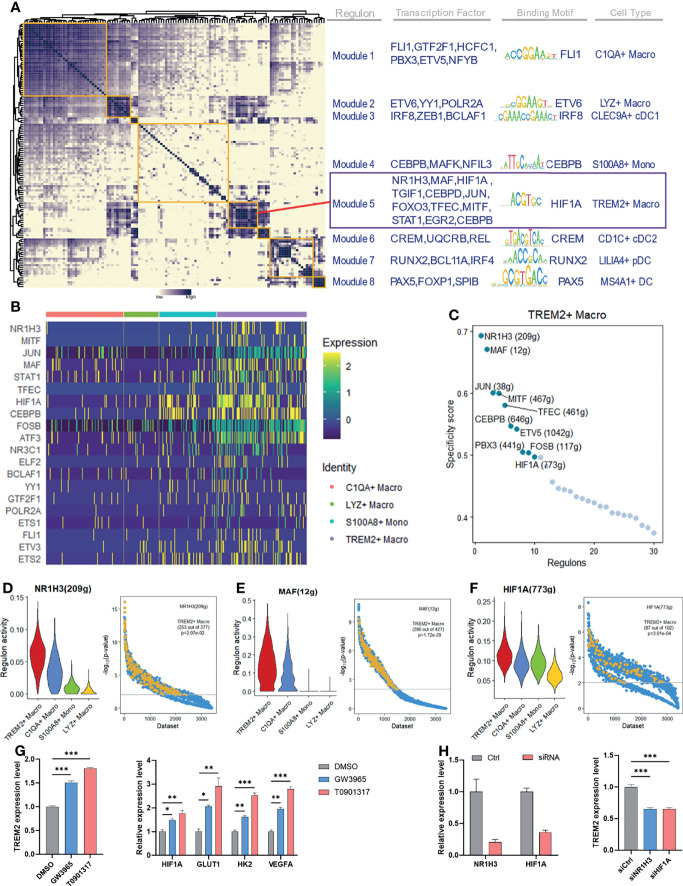
Identified specific regulators of the maintenance of TREM2^+^ macrophage identity. **(A)** Identification of regulon modules based on the regulon connection specificity index matrix in myeloid cells, along with representative transcription factors, corresponding binding motifs, and associated cell subtypes. **(B)** Heatmap showing the expression of the selected transcription factors across monocyte–macrophage subtypes. Columns denote cells; rows denote genes. **(C)** Rank of the regulons in *TREM2* ^+^ macrophages based on the regulon specificity score. The g within the bracket indicates genes. **(D–F)** Violin plots showing the regulon activity in *TREM2* ^+^ macrophages (left) and SEEK coexpression results for target genes of the indicated regulon in different GEO datasets. *TREM2* ^+^ macrophage-related datasets with a significant correlation (*P* < 0.01) are highlighted by yellow dots. **(G, H)** PMA-induced THP-1 cells were treated with LXR agonists **(G)** or siRNAs **(H)**. The relative expression levels of the indicated genes were determined by qRT-PCR. Significance was assessed using one-way ANOVA with Tukey’s *post hoc* test. *P < 0.01; **P < 0.01; ***P < 0.001.

To identify potential transcriptional regulators of TREM2^+^ LAM-like cells, we employed SCENIC to decipher the gene regulatory network modules specific to each myeloid subtype. Eight major modules were identified across myeloid cells, and *TREM2* ^+^ LAM-like cells were associated with module 5 (**Figure 6A**). We observed specific activation of the module-5-associated transcriptional factors *NR1H3* (LXR-α), *MAF*, and *HIF1A* in *TREM2* ^+^ LAM-like cells, but this activation was relatively absent in other monocyte–macrophage subsets (**Figure 6B**). We then ranked regulons in *TREM2* ^+^ LAM-like cells according to their specificity score, which was defined based on the Jensen-Shannon divergence (37). *NR1H3*, *MAF*, and *HIF1A* were identified by network analysis as the most specific regulons associated with *TREM2* ^+^ LAM-like cells (**Figure 6C**). To evaluate the accuracy of the regulon prediction, we applied SEEK analysis for GEO dataset data mining. The results indicated that the target genes in the *NR1H3*, *MAF*, and *HIF1A* regulons were significantly coexpressed in *TREM2* ^+^ macrophage-related datasets (**Figures 6D–F**). We next employed *in vitro* assays to test the assumption that *NR1H3* and *HIF1A* may facilitate the reprogramming of *TREM2* ^+^ LAM-like cells. Activation of LXR with the synthetic agonists GW3965 and T0901317 triggered a significant increase in *TREM2* expression levels in human macrophages. In addition, exposure to LXR agonists resulted in marked induction of *HIF1A* and downstream target genes related to hypoxia, glycolysis, and angiogenesis (*GLUT1*, *HK2*, and *VEGFA*; **Figure 6G**). In contrast, silencing *NR1H3* or *HIF1A* with siRNA significantly reduced *TREM2* expression (**Figure 6H**). Furthermore, analysis of publicly available datasets revealed that GW3965 augmented LXR-α binding peaks associated with the *Trem2* gene locus in mouse macrophages (**Supplementary Figure 4**). These results highlight the potential role of LXR signaling in the establishment of a specific transcription program in TREM2^+^ LAM-like cells.

### Infiltration of TREM2^+^ LAM-Like Cells Is Associated With Worse Prognosis

**Figure 7:**
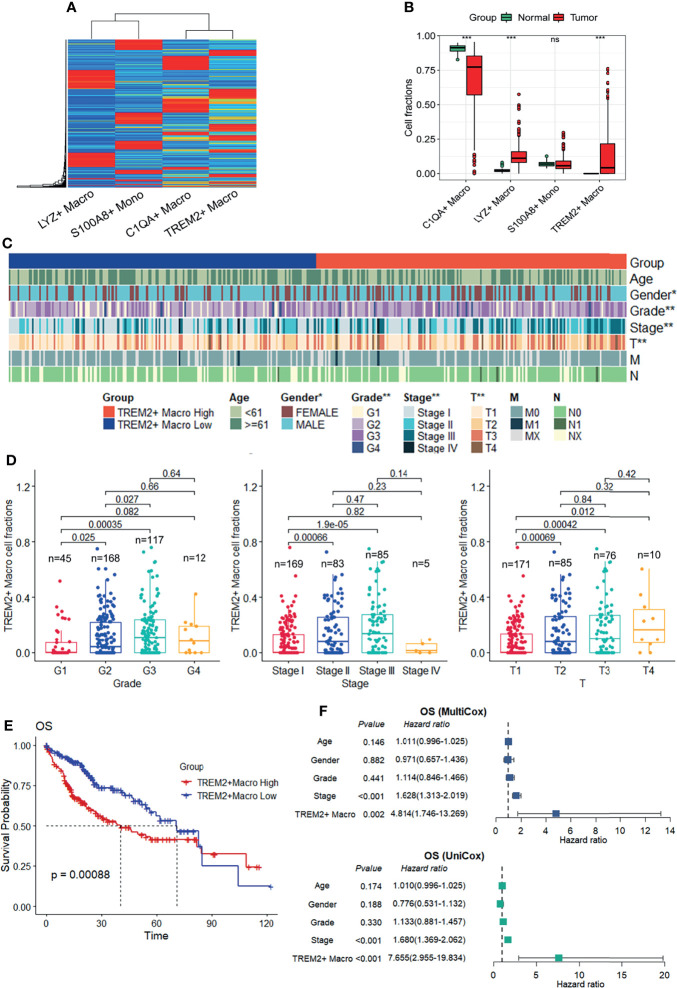
Prognostic role of the TREM2^+^ macrophage subtype in the TCGA-LIHC cohort. **(A)** The signature matrix created by CIBERSORTx. **(B)** Box plot showing the relative frequency of macrophage subtypes and monocyte infiltration calculated by CIBERSORTx with the default relative mode, which normalizes all cell fractions of the cell types in the signature matrix to 100%. Significance was determined by unpaired Wilcoxon test. **(C)** Strip chart showing the distribution of common clinical features, including tumor grade, AJCC pathologic stage, AJCC pathologic T, AJCC pathologic N, and AJCC pathologic M, in patients with a high or low fraction of *TREM2* ^+^ macrophages. *P < 0.05, **P < 0.01. **(D)** Frequency of *TREM2* ^+^ macrophages in patients with different tumor grades, pathologic stages, and T stages. *P* values were determined by chi-square tests. **(E)** Kaplan-Meier survival curves showing the overall survival (OS) of patients in the TCGA-LIHC cohort according to the frequency of *TREM2* ^+^ macrophages. *P*-values were calculated using log-rank tests. **(F)** Univariate (top) and multivariate (bottom) Cox hazard ratio analyses of OS.

To evaluate the relative abundance of the *TREM2* ^+^ LAM-like subtype and its clinical relevance in liver cancer patients, a signature matrix was first created from human HCC scRNA-seq data (**Figure 7A**) using CIBERSORTx (31), providing a reference atlas for the deconvolution of bulk RNA-seq profiles. We assessed the hepatic monocyte–macrophage composition in the TCGA-LIHC cohort and found that the fraction of infiltrating *TREM2* ^+^ LAM-like cells was significantly increased in tumor samples compared to normal tissues (**Figure 7B**). We also investigated the possible correlations between *TREM2* ^+^ macrophage frequency and clinical features. The strip chart showed that the high *TREM2* ^+^ macrophage group was significantly more likely to have advanced tumor grade, pathologic stage, and T stage (**Figure 7C**). On the other hand, patients with advanced tumor grade, pathologic stage, and T stage were significantly associated with increased accumulation of *TREM2* ^+^ LAM-like cells (**Figure 7D**). Notably, Kaplan-Meier survival analysis demonstrated an apparent association between a higher fraction of *TREM2* ^+^ LAM-like cells and unfavorable overall survival, progression-free interval, disease-free interval, and disease-specific survival in patients from TCGA-LIHC (**Figure 7E**, **Supplementary Figures 5A–C**). Moreover, univariate and multivariate Cox regression analyses indicated that *TREM2* ^+^ macrophage frequency was an independent prognostic factor for survival prediction after adjusting for clinical factors, including age, sex, grade, and stage (**Figure 7F**, **Supplementary Figures 5D–F**).

## Discussion

The heterogeneity of the tumor ecosystem considerably reduces the efficacy of clinical treatments and makes prognostic prediction quite complicated in HCC (38). In recent years, the research community has focused on the central role of myeloid cells in diverse pathologies, with TREM2 being highlighted as a major pathology-induced immune signaling hub (7). In this study, we analyzed scRNA-seq data to explore the expression pattern and function of TREM2 in human HCC. We defined a group of macrophages that highly express TREM2 with a proangiogenic phenotype and an immunosuppressive state. Although these *TREM2* ^+^ macrophages exhibited a transcriptome most similar to hepatic LAMs and were hence termed LAM-like cells, they share expression of a subset of genes associated with SAMs, consistent with the recent study (13). It is worth noting that the gene expression profile of *TREM2* ^+^ LAM-like cells also showed some overlap with that of Kupffer cells but lacked the expression of *TIMD4*, *MARCO*, *CD5L*, and *VCAM1*. Further work is required to better understand the overlap with LAMs/SAMs and Kupffer cells and the precise nature of these *TREM2* ^+^ LAM-like cells.

Functional enrichment analysis indicated the enrichment of angiogenesis, hypoxia, and glycolysis signaling pathways in *TREM2* ^+^ LAM-like cells. The results are consistent with a prior study showing that Trem2 promotes the transition of proinflammatory macrophages to restorative macrophages and the emergence of endothelial cells during recovery from liver injury (39). A *TREM2* loss-of-function mutation (T66M) impairs microglial function and causes a significant reduction in brain glucose metabolism (40). Indeed, TREM2-deficient microglia show decreased expression of glycolytic genes and impairment of cell energetic and biosynthetic metabolism in Alzheimer’s disease (41).

Intriguingly, ligand-receptor interaction analysis demonstrated that *TREM2* ^+^ LAM-like cells could mediate VEGF signaling to promote angiogenesis. *TREM2* ^+^ LAM-like cells were predicted to interact with endothelial cells using angiogenesis-related ligands and receptor pairs (VEGFA : KDR, VEGFA : NRP1, VEGFA : NRP2, VEGFA : FLT1, and VEGFB : NRP1) in the TME (**Supplementary Figure 6A**), in concordance with the single-cell atlas of cirrhotic human livers (12). We also identified the VEGF signaling pathway network *via* CellChat analysis and confirmed that *TREM2* ^+^ LAM-like cells were prominent sources of VEGF ligands interacting with receptors expressed on endothelial cells (**Supplementary Figure 6B**), suggesting the importance of *TREM2* ^+^ macrophage-mediated VEGF signaling in promoting angiogenesis in HCC.

TREM2 transmits intracellular signals in various contexts, leading to significant changes in cellular phenotypes and functions, including phagocytosis induction, lipid metabolism, and metabolic shift (7). Variation analysis of regulons with high expression levels between *TREM2* ^+^ LAM-like cells and other monocyte–macrophage subgroups illustrated that *NR1H3*, *HIF1A*, and *MAF* were potential transcriptional regulators of *TREM2* ^+^ LAM-like cells. In addition, our trajectory inference analysis showed that *TREM2^+^* LAM-like cells would derive from monocytes, consistent with the origination of *TREM2^+^* LAMs and SAMs (12–14). *NR1H3* (LXR-α) stimulation in macrophages differentiated from human monocytic THP-1 cells showed increased *TREM2*, *HIF1A*, and downstream target genes related to hypoxia, glycolysis, and angiogenesis, suggesting that the LXR-α signaling would facilitate the reprogramming of *TREM2* ^+^ LAM-like cells. Studies with primary human monocytes will further validate the role of LXR-α in acquiring *TREM2* and other genes associated with the LAM-like phenotype. In line with these results, LXR-α represents an essential link between cholesterol accumulation and negative regulation of the inflammatory response in macrophages (42). The cholesteryl ester accumulation caused by TREM2 deficiency in microglia can be reversed by the LXR agonist GW3965 (43). In addition, LXR-α activation can potentiate HIF-1α signaling and glycolysis in human macrophages (44), and there is a positive feedback circuit connecting the activation of HIF-1α and LXR-α in foam cell formation (45). Furthermore, *Maf*, *Atf3*, and *Hif1a* are potential regulators of regulatory myeloid (Mreg) cells, while *Hif1a* is a potential regulator of tumor-associated macrophages in MCA205 mouse tumors; these two myeloid subpopulations share the expression of Arg1 and Trem2 (Arg1^+^Trem2^+^) (46). It has been suggested that anti-inflammatory TREM2 signaling may interfere with intrinsic mechanisms to combat neoplasia and promote profibrotic responses following liver damage (7). The TREM2^+^CD9^+^ subset of macrophages that differentiate from circulating monocytes expands during liver cirrhosis and contributes to fibrosis (12). Moreover, a nonalcoholic steatohepatitis (NASH)-related diet induces the collaboration of *Atf3* with LXRs to induce *Trem2* and *Cd9* expression, promoting the establishment of the SAM and/or LAM phenotypes (47). Regulatory factors including *ATF3* (increased in *TREM2* ^+^ LAM-like cells; **Figure 6B**) might also collaborate with *NR1H3* to modulate the reprogramming of human *TREM2* ^+^ LAM-like cells. LXR-α,β*^Dko^* mice develop M1 macrophage-predominant chronic lung inflammation and eventually peripheral lung squamous cell carcinoma-like lesions (48). The precise delineation of mechanistic regulators is warranted to understand better how the *TREM2* ^+^ LAM-like cells are established in HCC.

TREM2 possesses immunosuppressive activities and promotes immune evasion in HCC. Survival analysis further showed that HCC patients with *TREM2* ^+^ macrophage enrichment in tumor tissues had significantly shorter survival. *TREM2* ^+^ macrophage frequency could serve as an independent predictor of prognosis, suggesting TREM2 as a promising therapeutic target to reverse the myeloid cell-derived immune-suppressive environment. In contrast, strategies employing agonistic compounds that enhance TREM2 signaling to boost the healing activities of macrophages and microglia are adopted for Alzheimer’s disease. Given that TREM2 was found to play a protective role in hepatocarcinogenesis in a mouse model, we hypothesize that TREM2^+^ LAM-like cells are a double-edged sword: they restrict inflammatory injury and tumorigenesis in the early stage but suppress the antitumor immune response and promote cancer progression in the later period.

In summary, our comprehensive characterization of TREM2^+^ LAM-like cells uncovered their potential as a novel prognostic biomarker and therapeutic target for HCC (**Supplementary Figure 7**). A further mechanistic understanding of TREM2 expression regulators and their downstream signaling pathways is expected to enable the development of effective immunotherapies targeting these myeloid mediators in cancers, including HCC.

## Data Availability Statement

The data sets analyzed during this study are available in public, open access repositories listed in this article.

## Ethics Statement

The studies involving human participants were reviewed and approved by the Ethics Committee of the First Affiliated Hospital, Shihezi University School of Medicine. The patients/participants provided their written informed consent to participate in this study.

## Author Contributions

LW and JH conceived the study. LZ, MW, HG, YZ, and ML searched and analyzed the data. LZ, MW, and HG wrote the draft of the paper. JH, XW, XC, and LW revised the manuscript. All authors have read and approved the final manuscript.

## Funding

This work was supported by the Science and Technology Cooperation Program of Xinjiang Production and Construction Corps (2021BC002), the Youth Science and Technology Innovation Leading Talents Project of Xinjiang Production and Construction Corps (2020CB015), the Youth Innovation Talents Project of Shihezi University (CXBJ201907), and the Non-profit Central Research Institute Fund of Chinese Academy of Medical Sciences (2020-PT330-003).

## Conflict of Interest

The authors declare that the research was conducted in the absence of any commercial or financial relationships that could be construed as a potential conflict of interest.

## Publisher’s Note

All claims expressed in this article are solely those of the authors and do not necessarily represent those of their affiliated organizations, or those of the publisher, the editors and the reviewers. Any product that may be evaluated in this article, or claim that may be made by its manufacturer, is not guaranteed or endorsed by the publisher.
