# Supplementary material for: Integrated Analysis Highlights the Immunosuppressive Role of TREM2+ Macrophages in Hepatocellular Carcinoma
Source: Front Immunol. 2022 Mar 14;13:848367. doi: 10.3389/fimmu.2022.848367 (PMC8963870; doi:10.3389/fimmu.2022.848367)
Supplement: Supplementary file 1 [file DataSheet_1.docx]

**Integrated analysis highlights the immunosuppressive role of TREM2^+^ macrophages in hepatocellular carcinoma**

Lisha Zhou, Meiling Wang, Hanrui Guo, Jun Hou, Yingna Zhang, Man Li, Xiangwei Wu, Xueling Chen, Lianghai Wang

**Table of Content**

Supplementary Figure 1

Supplementary Figure 2

Supplementary Figure 3

Supplementary Figure 4

Supplementary Figure 5

Supplementary Figure 6

Supplementary Figure 7

Supplementary Table 1

Supplementary Table 2

**
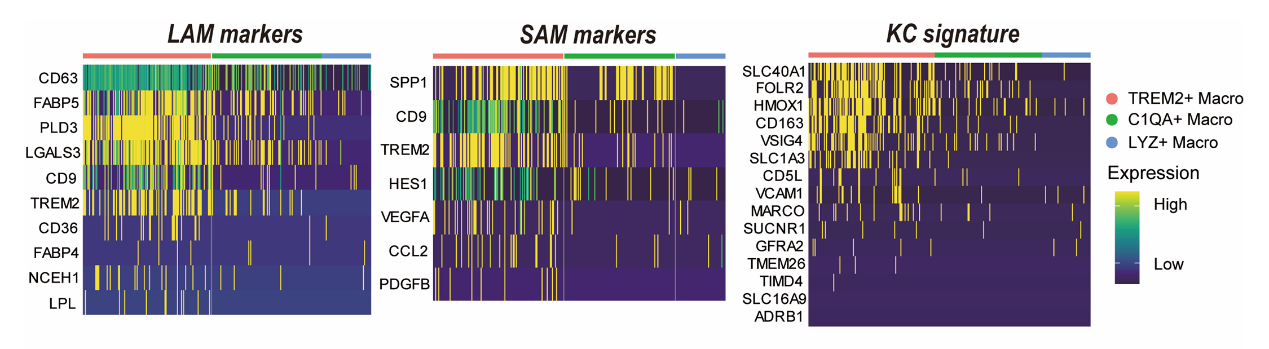
Supplementary Figure 1. Expression of macrophage signature genes across cell subtypes.** Genes enriched in LAM (left) and SAMs (middle) (1). Human-murine KC signature genes (right) (2).


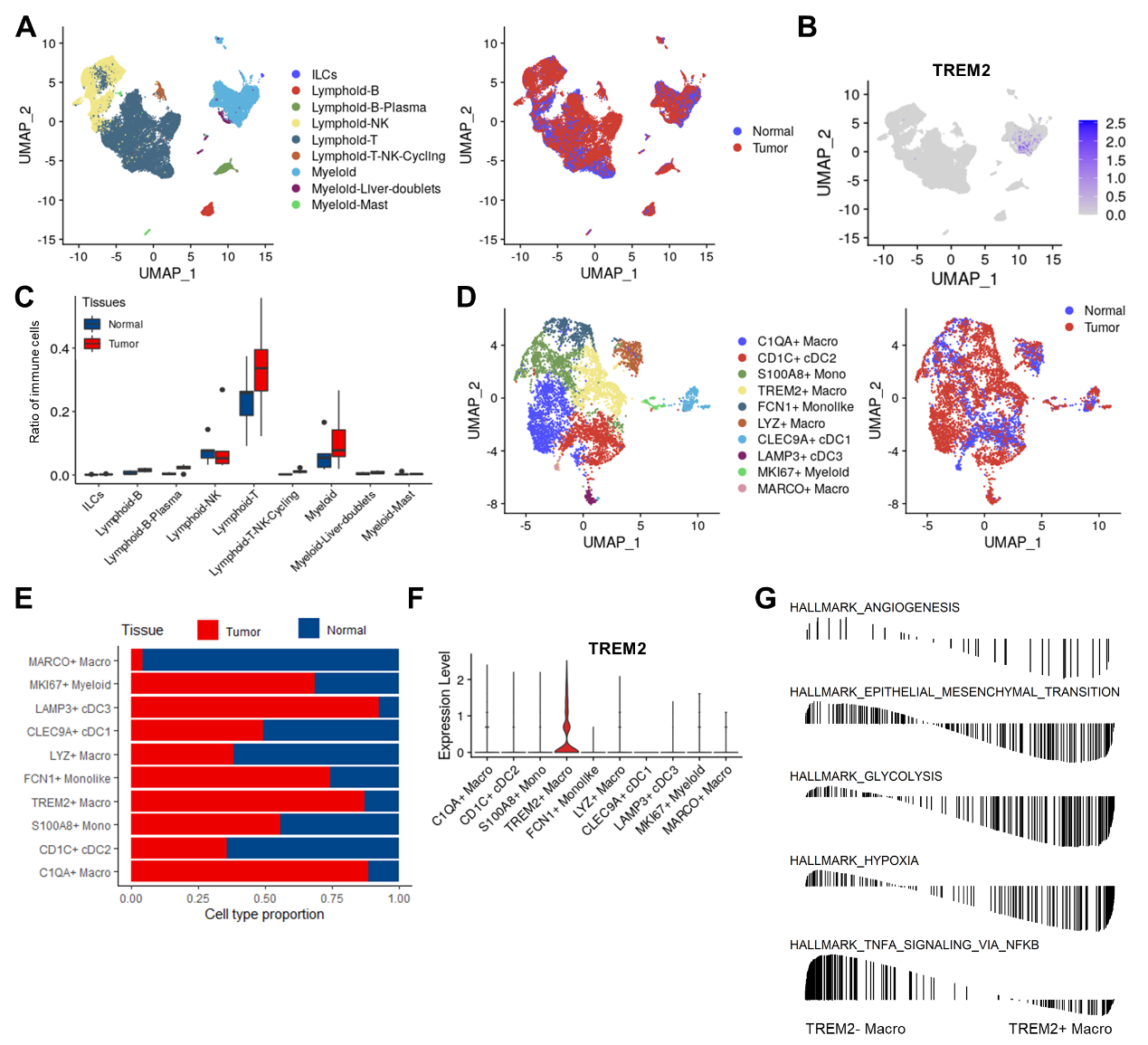
**Supplementary Figure 2. Identification of the tumor-infiltrating TREM2^+^ macrophage subtype in the validation cohort.** (A) UMAP projection showing the cell types of CD45^+^ immune cells based on scRNA-seq data from Validation cohort 1 colored by cluster (left) and tissue origin (right). (B) UMAP plot of CD45^+^ immune cells colored by *TREM2* expression level. (C) Box plot illustrating the fraction of immune cell types in normal and tumor tissues. (D) UMAP visualization of the subtypes of myeloid cells colored by cluster (left) and tissue origin (right). (E) Stacked histogram representing the proportion of tumor and normal tissue-derived cells in each myeloid subtype. (F) Violin plot showing the expression level of *TREM2* in myeloid subgroups. (G) Enrichment plots of significantly enriched hallmark gene sets in *TREM2*^+^ and *TREM2*^-^ macrophages.

**
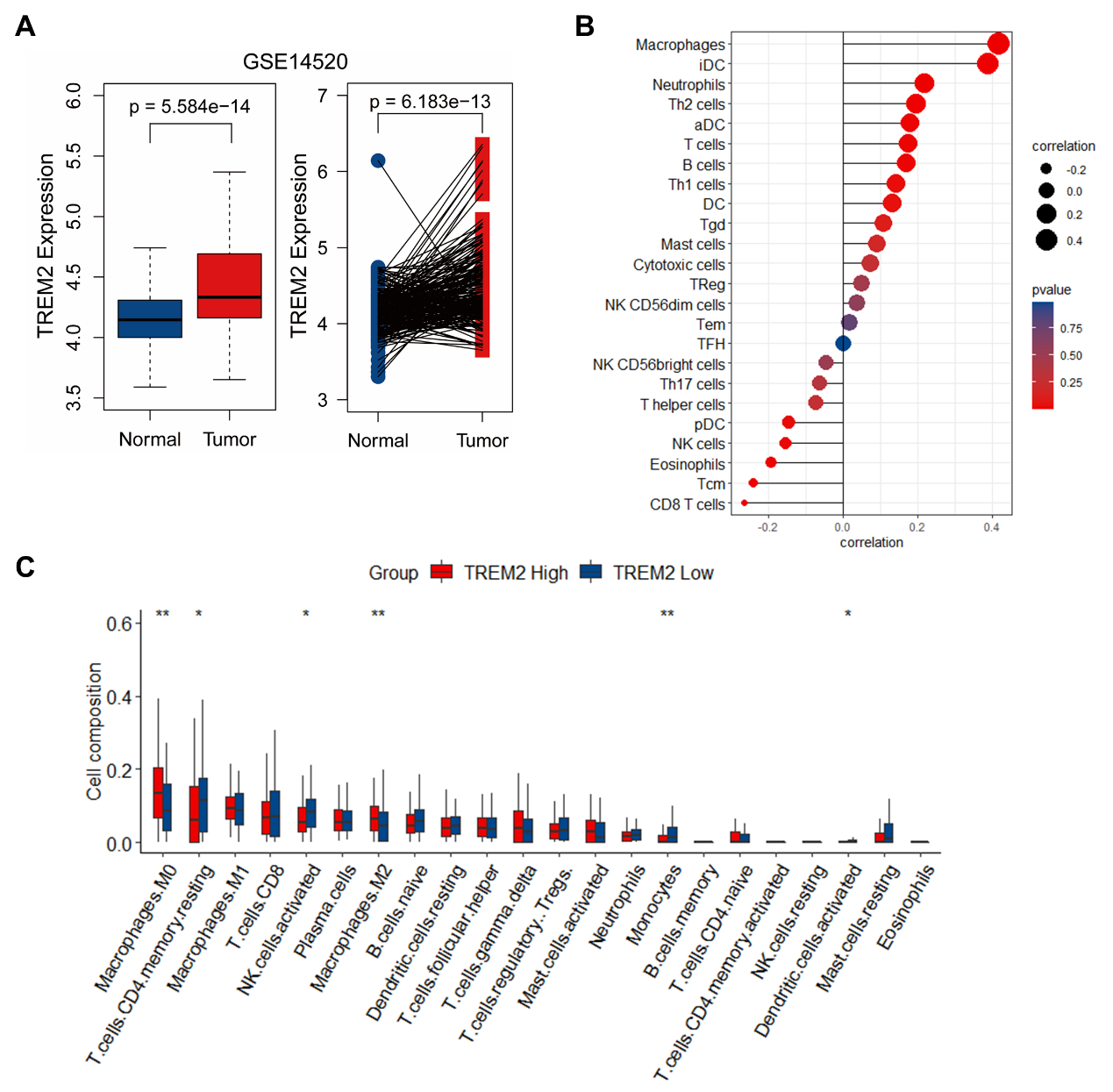
**

**Supplementary Figure 3. TREM2 is upregulated in HCC and correlates with macrophage infiltration.** (A) Box plots showing *TREM2* expression values in 225 HCC and 220 nontumor tissues (left) and 213 paired samples from the GSE14520 dataset. *P* values were determined by unpaired or paired Wilcoxon tests. (B) Lollipop plot depicting Spearman's correlation coefficients between *TREM2* expression and 24 immune cell types calculated by ssGSEA. (C) Box plot illustrating the infiltration of 22 immune cell types estimated by CIBERSORT in patients with high and low *TREM2* expression levels from the GSE14520 cohort. Significance was determined by unpaired Wilcoxon test. **P* < 0.05, ***P* < 0.01.

**
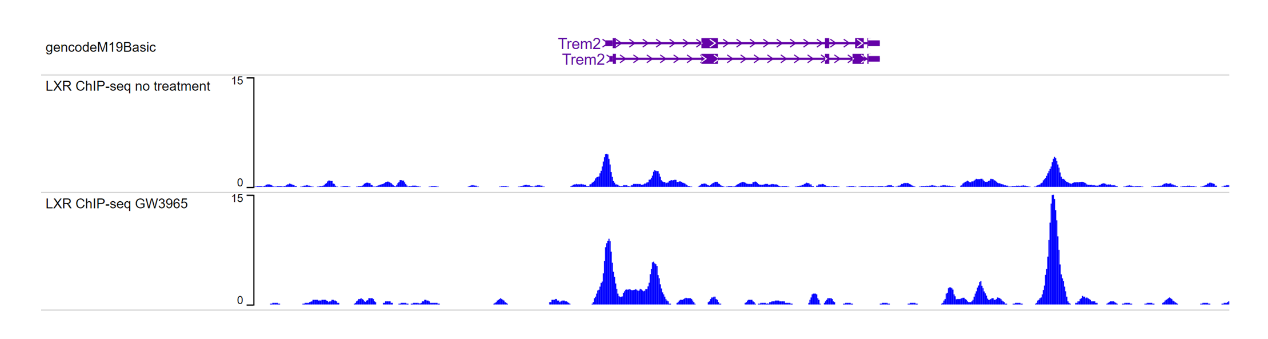
**

**Supplementary Figure 4.** Exploration and visualization of the LXR-α binding peaks on the *Trem2* locus in the GSE79423 dataset with Cistrome Data Browser (http://cistrome.org/db/#/).

**
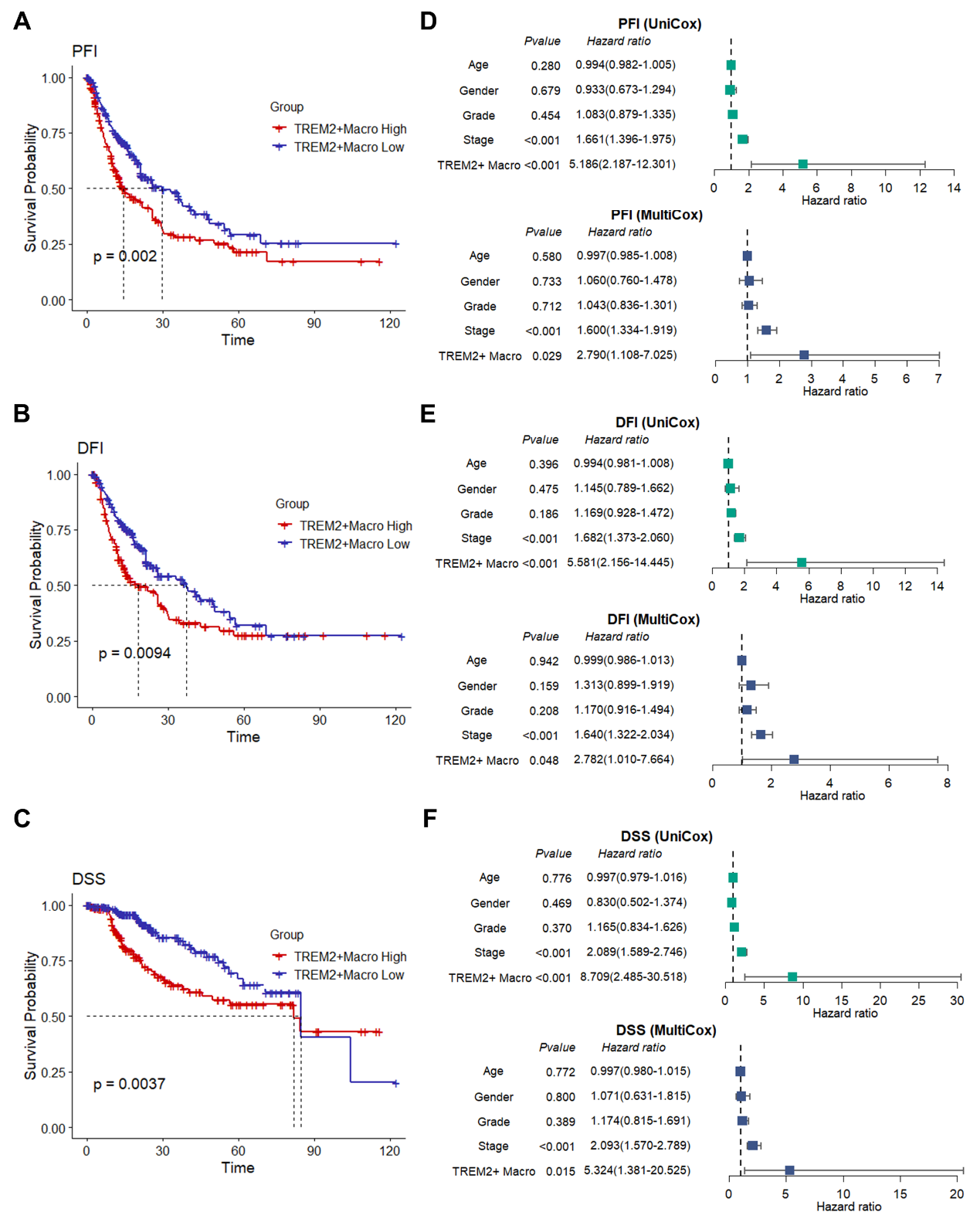
**

**Supplementary Figure 5. Prognostic utility of TREM2^+^ macrophage frequency in the TCGA-LIHC cohort.** (A–C) Kaplan-Meier survival curves showing the progression-free interval (PFI), disease-free interval (DFI), and disease-specific survival (DSS) of patients in the TCGA-LIHC cohort according to the relative abundance of *TREM2*^+^ macrophages. *P* values were calculated using log-rank tests. (D–F) Univariate (top) and multivariate (bottom) Cox hazard ratio analyses demonstrating that *TREM2*^+^ macrophage frequency is an independent survival predictor.

**
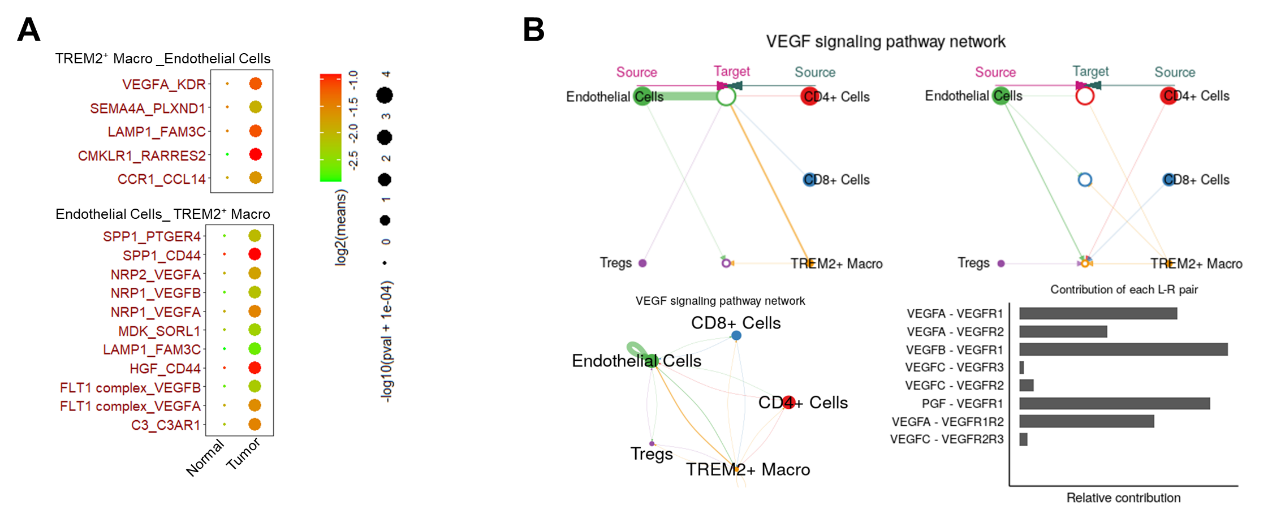
**

**Supplementary Figure 6. Potential interaction of TREM2^+^ macrophages with endothelial cells.** (A) Dot plot of HCC-specific ligand-receptor interactions of *TREM2*^+^ macrophages with endothelial cells. (B) Hierarchical plot providing an overview of the inferred VEGF signaling pathway network detected by CellChat. The left and right portions show the autocrine and paracrine signaling between cell groups (top). The number of significant ligand-receptor pairs between cell populations and the relative contribution of each ligand-receptor pair to the overall communication network (bottom) is shown.

**
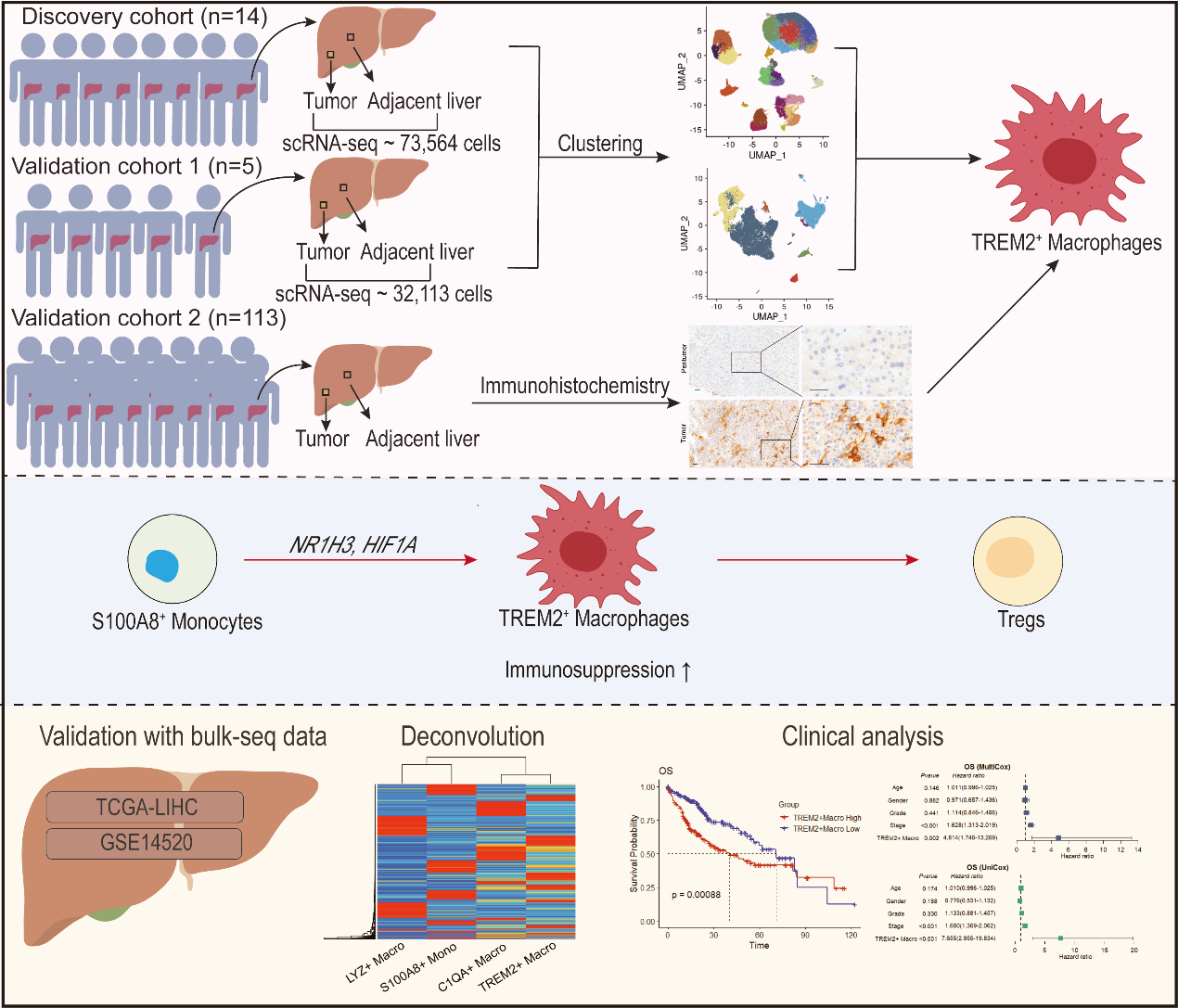
**

**Supplementary Figure 7. Schematic diagram for comprehensive characterization of the immunosuppressive role of TREM2^+^ macrophages in HCC.**

**References**

1. Daemen S, Gainullina A, Kalugotla G, He L, Chan MM, Beals JW, et al. Dynamic Shifts in the Composition of Resident and Recruited Macrophages Influence Tissue Remodeling in NASH. Cell Rep. 2021;34(2):108626.

2. Guilliams M, Bonnardel J, Haest B, Vanderborght B, Wagner C, Remmerie A, et al. Spatial proteogenomics reveals distinct and evolutionarily conserved hepatic macrophage niches. Cell. 2022;185(2):379-96 e38.

**Supplementary Table 1. Signature genes for GSVA.**

| Pathway | Signature genes |
| --- | --- |
| Angiogenesis | CCND2, CCNE1, CD44, CXCR4, E2F3, EDN1, EZH2, FGF18, FGFR1, FYN, HEY1, ITGAV, JAG1, JAG2, MMP9, NOTCH1, PDGFA, PTK2, SPP1, STC1, TNFAIP6, TYMP, VAV2, VCAN, VEGFA |
| Phagocytosis | *MRC1, CD163, MERTK, C1QB* |
| M1 | *IL23, TNF, CXCL9, CXCL10, CXCL11, CD86, IL1A, IL1B, IL6, CCL5, IRF5, IRF1, CD40, IDO1, KYNU, CCR7* |
| M2 | *IL4R, CCL4, CCL13, CCL20, CCL17, CCL18, CCL22, CCL24, LYVE1, VEGFA, VEGFB, VEGFC, VEGFD, EGF, CTSA, CTSB, CTSC, CTSD, TGFB1, TGFB2, TGFB3, MMP14, MMP19, MMP9, CLEC7A, WNT7B, FASL, TNFSF12, TNFSF8, CD276, VTCN1, MSR1, FN1, IRF4* |
| TCA cycle | *DLAT, DLD, PDHA1, CS, DLST, FH, IDH3A, MDH1, OGDH, SDHA, SUCLG2, PDHA2, PDHB, ACO2, IDH3G, MDH2, SDHB, SDHC, SDHD, SUCLG1, SUCLA2, ACLY, ACO1, IDH1, PC, PCK1, ALDOA, ALDOB, ALDOC, ENO2, HK1, HK3, PFKL, PFKM, PFKP, PGAM2, PGK1, PKLR, PKM, GAPDHS, PGAM4, IDH2, IDH3B, PCK2, OGDHL* |
| Lipolysis | *AKT3, ADCY1, ADCY2, CGA, ADCY3, ADCY5, PLAAT3, ADCY6, LOC112267859, ADCY7, MGLL, ADCY8, ADCY9, ADORA1, ADRB1, ADRB2, ADRB3, ADCY4, AKT1, AKT2, FABP4, GNAI1, GNAI2, GNAI3, GNAS, INS, AQP7, INSR, IRS1, LIPE, NPY, NPPA, NPR1, NPY1R, ABHD5, PDE3B, PIK3CA, PIK3CB, PIK3CD, PIK3R1, PIK3R2, PLIN1, PRKACA, PRKACB, PRKACG, PRKG1, PRKG2, PNPLA2, PTGER3, PTGS1, PTGS2, TSHB, TSHR, IRS4, PIK3R3, IRS2* |
| Glutamine metabolism | *GLS, GLS2, GLUD1, GLUD2, DGLUCY* |

**Supplementary Table 2. Oligonucleotide sequences for gene knockdown.**

| Symbol | Sequence |
| --- | --- |
| siHIF1A | sense: GCCGCUCAAUUUAUGAAUATT |
|  | antisense: UAUUCAUAAAUUGAGCGGCTT |
| siNR1H3 | sense: CACGGAUGCUAAUGAAACUTT |
|  | antisense: AGUUUCAUUAGCAUCCGUGTT |
